# Comparing the infection biology and gene expression differences of *Plasmodiophora brassicae* primary and secondary zoospores

**DOI:** 10.3389/fmicb.2022.1002976

**Published:** 2022-12-01

**Authors:** Hui Yang, Qianyu Sun, Yihan Zhang, Yang Zhang, Yushan Zhao, Xinyue Wang, Yanmei Chen, Shu Yuan, Junbo Du, Wenming Wang

**Affiliations:** ^1^College of Agronomy, Sichuan Agricultural University, Chengdu, China; ^2^National Demonstration Center for Experimental Crop Science Education, College of Agronomy, Sichuan Agricultural University, Chengdu, China; ^3^College of Resources, Sichuan Agricultural University, Chengdu, China; ^4^State Key Laboratory of Crop Gene Exploration and Utilization in Southwest China, Sichuan Agricultural University, Chengdu, China

**Keywords:** clubroot, *P. brassicae*, infection biology, differentially expressed gene, primary zoospores, secondary zoospores

## Abstract

*Plasmodiophora brassicae* (Wor.) is an obligate plant pathogen affecting Brassicae worldwide. To date, there is very little information available on the biology and molecular basis of *P. brassicae* primary and secondary zoospore infections. To examine their roles, we used microscope to systematically investigate the infection differences of *P. brassicae* between samples inoculated separately with resting spores and secondary zoospores. The obvious development of *P. brassicae* asynchrony that is characterized by secondary plasmodium, resting sporangial plasmodium, and resting spores was observed at 12 days in *Brassica rapa* inoculated with resting spores but not when inoculated with secondary zoospores at the same time. Inoculation with resting spores resulted in much more development of zoosporangia clusters than inoculation with secondary zoospores in non-host *Spinacia oleracea*. The results indicated that primary zoospore infection played an important role in the subsequent development. To improve our understanding of the infection mechanisms, RNA-seq analysis was performed. Among 18 effectors identified in *P. brassicae*, 13 effectors were induced in *B. rapa* seedlings inoculated with resting spores, which suggested that the pathogen and host first contacted, and more effectors were needed. Corresponding to those in *B. rapa*, the expression levels of most genes involved in the calcium-mediated signaling pathway and PTI pathway were higher in plants inoculated with resting spores than in those inoculated with secondary zoospores. The ETI pathway was suppressed after inoculation with secondary zoospores. The genes induced after inoculation with resting spores were suppressed in *B. rapa* seedlings inoculated with secondary zoospores, which might be important to allow a fully compatible interaction and contribute to a susceptible reaction in the host at the subsequent infection stage. The primary zoospores undertook an more important interaction with plants.

## Introduction

The clubroots caused by *Plasmodiophora brassicae* Woronin represent an emerging threat to Cruciferae crop production in many countries (Dixon, 2014). The disease cycle consists of a primary phase in the root hairs followed by a secondary phase in the root cortex. In the soil, resting spores germinate and release primary zoospores to penetrate the root hairs. Within the root hairs, *P. brassicae* forms zoosporangia that produce secondary zoospores (Dobson and Gabrielson, 1983). This process takes approximately 5 days at the optimum temperature (Sharma et al., 2011). Secondary zoospores are released into the soil and the lumen of root epidermal cells to infect root cortical cells and develop into secondary plasmodia. Secondary zoospores are also able to reinfect healthy root hairs and repeat the zoosporangial stage in root hairs and epidermal cells (Naiki et al., 1984). Finally, the plasmodia divide to form long-lived resting spores, marking the completion of a life cycle (Kageyama and Asano, 2009; Liu et al., 2020a).

The role of root hair infection in subsequent cortical infection and development is still not well-understood. Inoculation with resting spores initially produces only primary (root hair) infection, but inoculation with secondary zoospores results in both primary and secondary infections in susceptible hosts. Primary infection occurs in both susceptible and resistant host cultivars and many non-host species. The secondary infection phase only continues to be complete in susceptible hosts but is unable to be completed in resistant hosts and non-hosts (Liu et al., 2020b). To date, the relationship between primary infection and secondary infection is still unclear, and we do not know how primary infection affects the initiation of resistance in host species.

Primary and secondary zoospores cannot be distinguished visually. Several studies have attempted to examine the primary and secondary stages separately. McDonald et al. (2014) found that inoculation with secondary zoospores alone resulted in lower disease severity in susceptible hosts than inoculation with resting spores alone. Feng et al. indicated that inoculation of non-host ryegrass with secondary zoospores resulted in secondary infection, but no clubs developed. In contrast, some root hairs were infected, and no secondary infection was detected in non-host ryegrass plants grown in infested soil (Feng et al., 2012). These results imply that recognition of infection by the host and initiation of a host response (e.g., induction or suppression of resistance) mainly occur during the primary infection.

The role of primary or secondary zoospore infection in the pathogenesis of *P. brassicae* is not fully understood. The mechanism through which *P. brassicae* primary or secondary zoospores overcome plant immunity to achieve successful infection is not well-understood. To examine the primary and secondary stages separately, inoculation studies with resting spores (source of primary zoospores) and secondary zoospores were conducted on a host cabbage (*Brassica rapa*) and a non-host spinach (*Spinacia oleracea*).

Plant pathogens have evolved a repertoire of effector proteins. To manipulate plant defenses and enable parasitic colonization, many plant pathogens deliver effector proteins into host cells to suppress plant immunity or cause changes in plant morphology that can increase infection successfully (Vleeshouwers and Oliver, 2014). Identifying the proteins that comprise the secretome of *P. brassicae* is an important step to discover the infection response (Rolfe et al., 2016). Recent studies identified some effectors during *P. brassicae* early infection at 3 days post-inoculation (dpi) and late infection at 17, 20, and 24 dpi (Chen et al., 2019; Pérez-López et al., 2020). However, no study has focused on comparing the effector proteins released by primary zoospores and secondary zoospores.

It is unclear why root hair infection of primary zoospores is a necessary step before cortical infection. Although the transcription data showed the gene expressions during the early and late stages of *P. brassicae* infection (Siemens et al., 2006; Päsold et al., 2010), no study compared the differences in host gene expressions between primary and secondary zoospore infections. For a better understanding of the host response and molecular basis of *P. brassicae* infections, our experiments were conducted to separately compare the transcriptional expression differences after inoculation with *P. brassicae* primary zoospores and secondary zoospores. Our study may contribute to understanding the cellular and molecular mechanisms underlying host resistance in clubroots. Elucidation of the mechanisms underlying *P. brassicae* pathogenesis and the host response would contribute to the development of novel strategies for managing clubroot disease.

## Materials and methods

### Preparation and inoculation of resting spores

*Plasmodiophora brassicae* was isolated from Dayi in Sichuan Province, China. The isolates were identified as pathotype 4 based on the differentials of Williams (1966), which were previously described (Zheng et al., 2019). Resting *P. brassicae* spores were prepared as previously described (Ji et al., 2014). Root galls were homogenized using 10% (m/v) sucrose in a blender. The slurry was filtered through eight layers of gauze, and the suspension was clarified by centrifugation at 3,000 × *g* for 10 min. The resting spore precipitate was suspended in sterile water, adjusted to a concentration of 1 × 10^7^ (spores)/ml, and stored at 4°C.

Seeds of a susceptible host cabbage (*Brassica rapa*) and non-host spinach (*Spinacia oleracea*) were placed on Petri dishes to germinate for 3–4 days in a plant growth chamber at 25°C under a light-dark cycle of 14:10 h. The germinations were then transferred into tubes containing Hoagland solution and incubated for 3–4 days. When the first euphylla was available, the seedlings were transferred into Hoagland solution containing 1 × 10^5^ (resting spores)/ml in a tube, and it was ensured that the roots were immersed in the resting zoospore suspension. The tube was packaged with adhesive black tape. The inoculated plants were maintained in a growth chamber under 14-h light/10-h dark cycles and a temperature of 25°C. Each of the inoculations was repeated in triplicate (more than 10 seedlings per replicate).

### Preparation and inoculation of secondary zoospores

To observe secondary zoospore infection in host and non-host plants, secondary zoospores were produced using a previous method (Feng et al., 2012; McDonald et al., 2014). Spinach seeds were planted in the soil. Seven-day-old spinach seedlings were inoculated with 1 × 10^7^ (resting spores)/ml under 14-h light/10-h dark cycles at a temperature of 25°C. At 5 dpi (days post-inoculation), the spinach (*Spinacia oleracea*) seedlings were uprooted, and the roots were washed with tap water. The roots were cut off and rinsed three times by shaking at 150 rpm for 20 min in distilled water. Then, the roots were shaken in 50 ml of distilled water at 100 rpm for 24 h to stimulate the release of secondary zoospores. The zoospore suspension was concentrated by centrifugation at 5,000 × *g* for 5 min, adjusted to 1 × 10^5^ (spores)/ml, and used immediately for inoculation. The zoospore suspension was transferred to a sterilized tube. The tube was packaged with adhesive black tape. Three 7-day-old cabbage or spinach seedlings were inserted into the tube, and it was ensured that the roots were immersed in the secondary zoospore suspension. The inoculated plants were maintained in a growth chamber under 14-h light/10-h dark cycles at a temperature of 25°C. At 4, 7, or 12 dpi, more than 10 seedlings were investigated. The inoculations were repeated three times.

To analyze the pathogenicity of the secondary zoospores, non-host spinach (*S. oleracea*) seedlings were inoculated with resting spores as described earlier. The infected spinach seedlings were dug out 5 days after the initial date of inoculation with resting spores. The spinach seedling roots were washed with tap water to thoroughly remove the resting spores from the root surfaces and then planted directly into the soil to provide a source of secondary zoospores. Five spinach seedlings infected with cabbage seedlings were planted together. The seedlings were maintained under 14-h light/10-h dark cycles at a temperature of 25°C. At 40 dpi, all of the seedlings were harvested, and the symptoms of 50 cabbage seedlings were determined.

### Morphological characterization

After dyeing with 0.5% fluorescent pink dye for 1 min, the background color of the roots on the slides was washed with ddH_2_O. The plasmodia, zoosporangia, and resting spores of *P. brassicae* were stained red. All infection stages were observed with a Carl Zeiss Microscope (GmbH37081, Göttingen, Germany).

### Analysis of RNA sequencing

The roots of 50 seedlings inoculated with resting spores were harvested at 4 dpi and 7 dpi, and the samples were named P-Y4 and P-Y7, respectively. The roots of 50 seedlings that were inoculated with secondary zoospores were harvested at 4 dpi, and the sample was named S-Q. The inoculation methods for the resting spores and secondary spores and the growth conditions of seedlings are described earlier. The roots were washed thoroughly with distilled water to remove the spores adsorbed onto the surface. They were then frozen in liquid nitrogen and stored at −70°C.

Total RNA was extracted from two biologically different root samples using TRIzol^®^ reagent (Life Technologies, Carlsbad, CA, USA) following the manufacturer’s protocol. The RNA quantities and qualities were determined using a 2100 BioAnalyzer (Agilent Technologies, Santa Clara, CA, USA). RNA libraries were constructed with 2 μg of total RNA and subjected to high-throughput sequencing with a HiSeq-4000 sequencer (Illumina, San Diego, CA, USA). The sequencing reads were compared to the reference databases of the *P. brassicae* genome^1^ and *B. rapa* genome (see text footnote 1), and the length statistics of the reference transcriptome sequences were determined with per scripts (Trapnell et al., 2009). The raw RNA-seq data were uploaded to the NCBI under accession ID SRP392270.

Raw reads from the sequencing machine were generated by Base Calling and saved in FASTQ format. Clean reads were generated by removing reads with adaptors, reads where the number of unknown bases was more than 10%, and low-quality reads (the percentage of the low-quality bases with which value ≤ 5 was more than 50% in one read). Two biological replicates of each sample, showing a correlation coefficient value of ≥ 0.95, were considered for subsequent gene expression analysis. Eight RNA libraries were analyzed by using the RNA-Seq approach and comparative differential expressed gene (DGE) profiling analysis (Wang et al., 2010). The fragments per kilobase million methods were used to calculate the normalized expression data of each gene (Mortazavi et al., 2008).

Differentially expressed genes (DEGs) were selected by the DEseq2 software, with the criteria of absolute Log2 (fold change) ≥ 1 or Log2 (fold change) ≤ −1 and false discovery rate ≤ 0.01 between two samples (Wang et al., 2010). To elucidate the metabolic pathways of these predicted genes, the Kyoto encyclopedia of genes and genomes (KEGG) pathways of DEGs were analyzed as the method reported previously (Wu et al., 2006). The R language software TBtools was used for the generation of the heatmap.

### Assessment of secretory activities directed by signal peptides from putative secretory proteins in yeast

A yeast signal sequence trap assay was adopted to assess the secretory activities directed by signal peptides (Jacobs et al., 1997). DNA extraction of *P. brassicae* was performed using a previous method (Zheng et al., 2019). The DNA sequences encoding signal peptides were amplified by a high-fidelity DNA polymerase, and the purified DNA was then cloned into the linearized pSUC2T7M13ORI plasmid. The recombinant vectors were transformed into the chemicompetent *Saccharomyces cerevisiae* yeast strain, YTK12. Positive clones were used to assess the secretory activities using growth assays. Positive clones were confirmed through PCR with the primer pair, pSUC2F/pSUC2R (Supplementary Table 2), and were streaked on CMD-W (0.67% yeast N base without amino acids, 0.075% tryptophan dropout supplement, 2% sucrose, 0.1% glucose, and 2% agar) plates and YPRAA (1% yeast extract, 2% peptone, 2% raffinose, and 2 μg/mL antimycin A) plates and used a 2,3,5-triphenyltetrazolium chloride (TTC) assay according to a previous study (Chen et al., 2019).

## Results

### The pathogenicity of secondary zoospores from non-hosts

**FIGURE 1:**
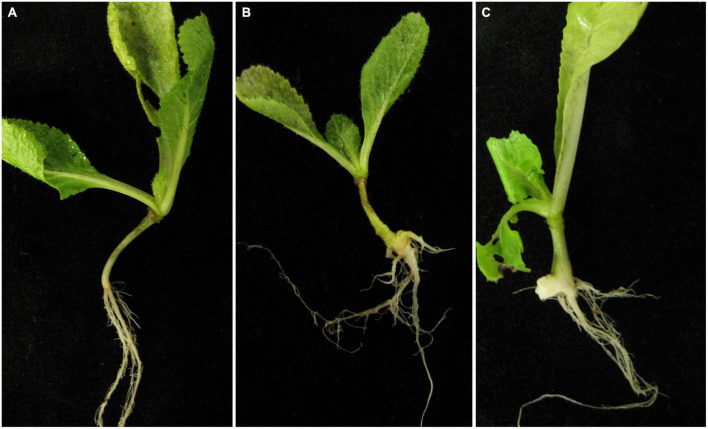
Symptoms of cabbage roots inoculated with primary and secondary zoospores. **(A)** Control, **(B)** symptoms of cabbage roots with secondary zoospore inoculation after 40 days, and **(C)** symptoms of cabbage roots with primary zoospore inoculation after 40 days.

Because secondary zoospores of different origins showed different infection abilities (Feng et al., 2012), we first tested the pathogenicity of secondary zoospores from non-host Spinacia. Spinach seedlings inoculated with resting spores were infected, and numerous zoosporangia developed in the root hairs. Healthy cabbage seedlings were planted with the infected spinach, which was used as an infection source of secondary zoospores. The cabbage roots showed obvious clubroot symptoms at 40 dpi; however, inoculation with secondary zoospores resulted in lower disease severity than inoculation with resting spores (Figure 1). The results showed the pathogenicity of secondary zoospores originating from the non-host spinach. No clubs were found on the spinach roots, regardless of whether they were inoculated with resting spores or secondary spores. Therefore, we next compared the infection biology after inoculation with secondary zoospores and primary zoospores.

### Infection biology of the host inoculated with primary and secondary zoospores

**FIGURE 2:**
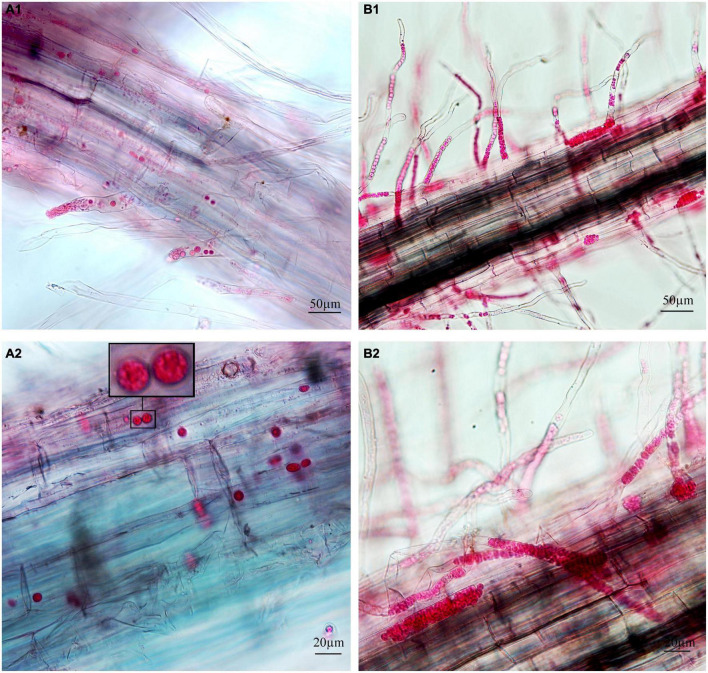
Infection of cabbage after inoculation with secondary zoospores. **(A1,A2)** Images of secondary zoospore inoculation after 7 days. **(B1,B2)** Images of secondary zoospore inoculation after 12 days.

**FIGURE 3:**
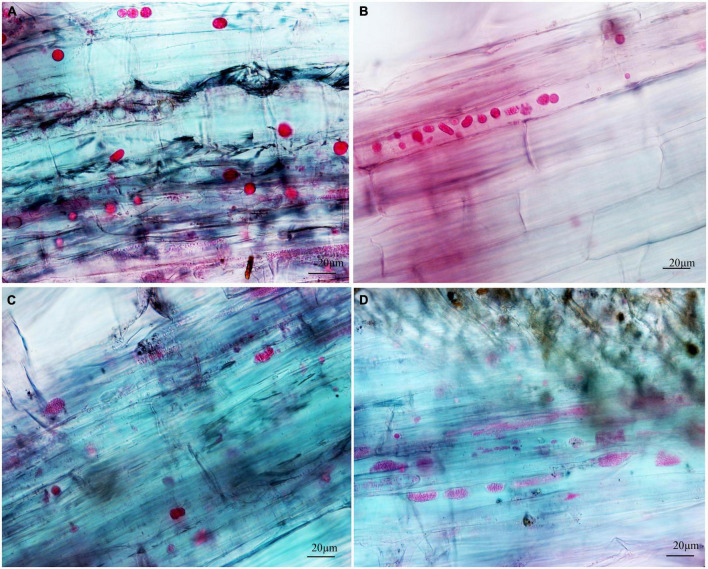
The developmental asynchrony of *P. brassicae* in cabbage inoculated with resting spores at 12 dpi. **(A)** The spherical secondary plasmodia with a visible membrane boundary. **(B)** The oblong and amorphous secondary plasmodia with a clear or unclear visible membrane boundary. **(C)** The resting sporangial plasmodia. **(D)** The resting sporangial plasmodia with no clear visible membrane boundary and many resting spores.

To separately examine the roles of primary and secondary zoospores in the host, inoculation studies using resting spores (source of primary zoospores) and secondary zoospores were conducted. At 7 days in cabbage inoculated with secondary zoospores, limited numbers of zoosporangia were detected, and at 12 dpi, the zoosporangia numbers gradually increased in the root hairs and root epidermal cells (Figure 2). However, at 12 days in cabbage inoculated with resting spores (namely, at 7 days after secondary zoospore inoculation), the developmental asynchrony of *P. brassicae* was obvious. The polymorphism was characterized by secondary plasmodium, resting sporangial plasmodium, and resting spores (Figure 3). Multinucleate secondary plasmodia that were spherical, compact, and separated from each other were observed, which were more organized with clearly visible membrane boundaries (Figure 3A). Many oblong or amorphous plasmodia were observed in most of the intracellular spaces (Figures 3B,C). A vast number of resting spores had formed (Figure 3D). Given that inoculation with *P. brassicae* resting spores leads to morphological variations, it is more asynchronous than inoculation with secondary zoospores, and we suggest that the primary zoospores present during root hair or epidermal cell infection can more efficiently affect host resistance.

### Infection biology of the non-host inoculated with primary and secondary zoospores

**FIGURE 4:**
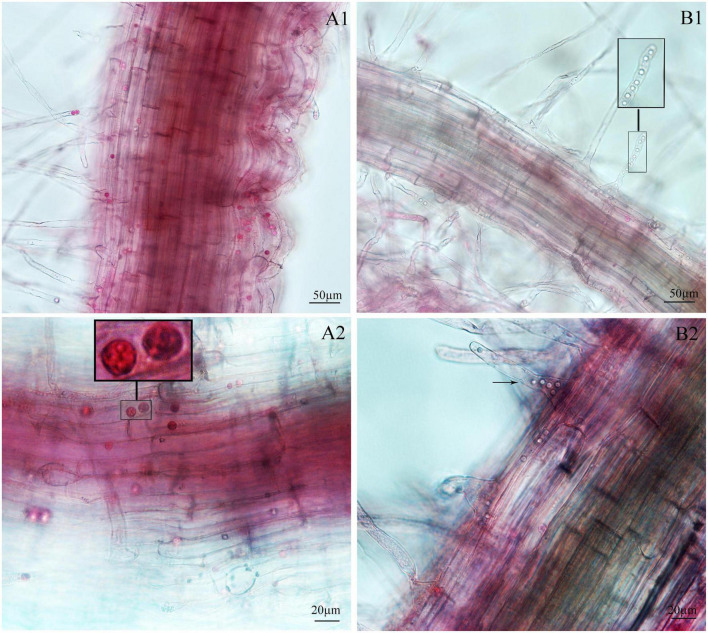
Infection of spinach inoculated with secondary zoospores. **(A1,A2)** Images of secondary zoospore inoculation after 7 days. **(B1,B2)** Images of secondary zoospore inoculation after 12 days.

**FIGURE 5:**
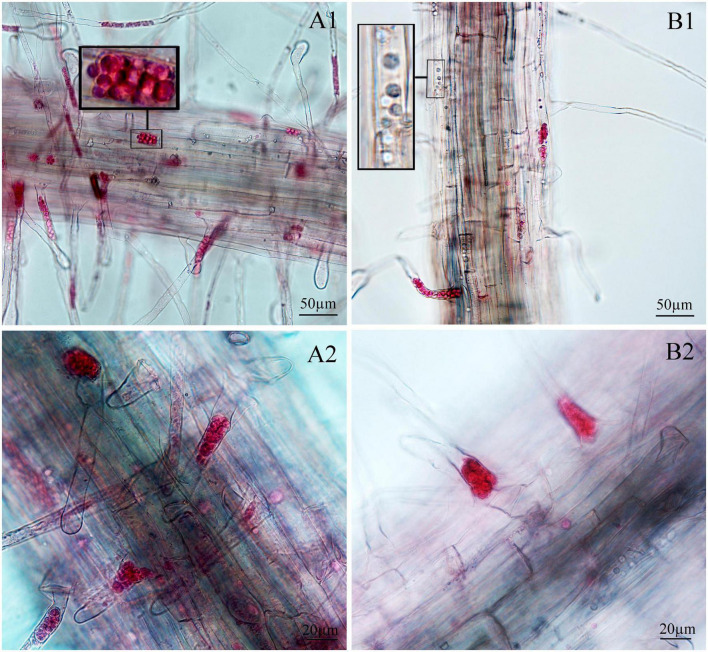
Infection of spinach inoculated with resting spores. **(A1,A2)** Images of resting spore inoculation after 12 days. **(B1,B2)** Images of resting spore inoculation after 17 days.

Next, we examined whether there were differences between the primary zoospore and secondary zoospore infections in non-host spinach. Inoculation studies using resting spores and secondary zoospores were conducted separately. At 7 and 12 days post-inoculation with secondary zoospores (namely, after the time of resting spore inoculation for approximately 12 and 17 days, respectively), zoosporangia were observed within the root hairs and epidermal cells, but we detected no zoosporangial clusters (Figure 4). However, at 12 and 17 days after inoculation with resting zoospores, zoosporangia clusters were observed (Figure 5). In spinach inoculated with resting spores, the proportion of zoosporangia per field of view in the microscope was higher than that with secondary zoospores. The results showed that inoculation with primary zoospores has a higher capability of zoosporangia proliferation than inoculation with secondary zoospores. Empty zoosporangia gradually appeared in seedlings inoculated with resting spores or secondary zoospores, indicating the release of secondary zoospores (Figures 4, 5).

### Identification of secreted proteins after inoculation by primary and secondary zoospores

Secretory proteins play critical roles in pathogen recognition and interactions among pathogens and hosts. In a previous study, we predicted some effector proteins based on the *P. brassicae* genome sequence in the NCBI. Here, to explore the possible reasons for the differences in infection biology, we compared the effector proteins in host plants that were inoculated with resting spores and secondary zoospores.

**FIGURE 6:**
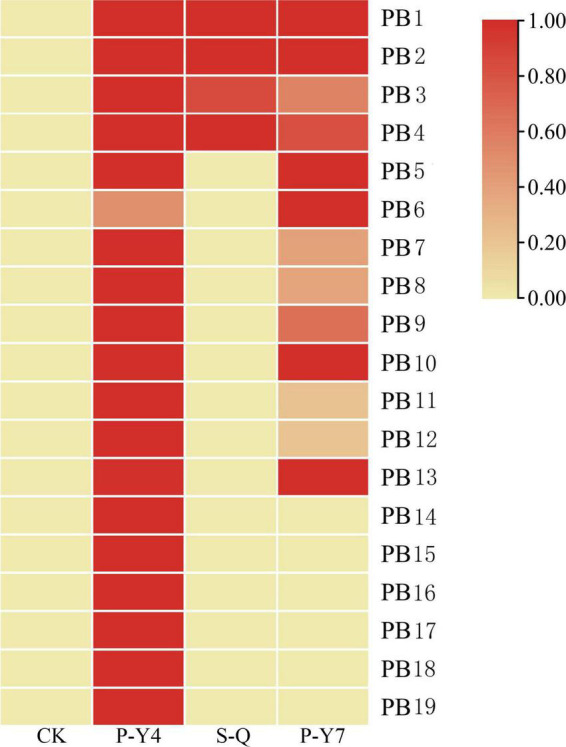
Heatmaps of *P. brassicae* effector expressions after primary zoospore and secondary zoospore inoculation. CK represents the control, P-Y4 represents effector expression at 4 days after resting spore inoculation, S-Q represents effector expression at 4 days after secondary zoospore inoculation, and P-Y7 represents effector expression at 7 days after resting spore inoculation.

Differentially expressed genes with fragments per kilobase million (FPKM) values greater than 5 were selected. Among the 19 putative secretory proteins, four genes (e.g., Pb1–Pb4) were upregulated in three samples (namely, two samples at 4 and 7 days after resting spore inoculation and one sample at 4 days after secondary zoospore inoculation). The expression levels of nine effector genes (e.g., Pb5–Pb13) were induced at both 4 and 7 days, and six effector genes (e.g., Pb14–Pb19) had high expressions only at 4 days after inoculation by primary zoospores (Figure 6). No putative secretory proteins were upregulated in the samples that were inoculated only with secondary zoospores at 4 days. The expressions of most effector genes were upregulated after inoculation with primary zoospores. The results indicate that primary zoospores may play a more important role in the host-*P. brassicae* interaction.

**FIGURE 7:**
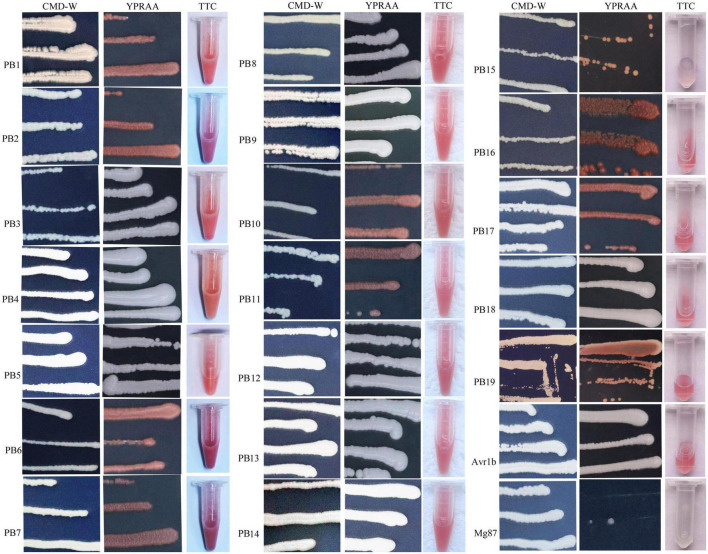
Functional validation of signal peptides from the putative secretory proteins of *P. brassicae*. YTK12 transformed with the corresponding pSUC-based construct was grown in CMD-W (proving the ability to produce tryptophan) and YPRAA (proving the ability to export invertase to the media). SPs from *Phytophthora sojae* Avr1b were used as the positive control, and *Magnaporthe oryzae* Mg87 served as the negative control.

The secretory activities directed by the signal peptides (SPs) of the above 19 putative effectors were tested. The primer sequence was listed in Supplementary Table 1. Eighteen SP sequences showed secretory activities, as demonstrated by growth assays on YPRAA and CMD-W plates in yeast and by the reduction of TTC to form insoluble red-colored 1,3,5-triphenylformazan (TPF) (Figure 7). Only Pb15, a predicted SP, did not demonstrate invertase activity in the chemicompetent *Saccharomyces cerevisiae* yeast strain, YTK12. Yeast transformed with Pb15 was unable to grow on YPRAA or reduce TCC.

### Host defense responses after inoculation by primary and secondary zoospores

Because the expression levels of many effectors were upregulated by primary zoospores, we next analyzed the host responses using RNA-seq data from root tissues of the resting spore and secondary zoospore inoculations. Approximately 57.84∼61.35 million raw RNA-Seq reads were produced from each sample. After filtering the dirty reads, 55.43∼58.71 million clean sequences were acquired and 70.10∼77.53% of sequences were mapped to the reference genome of *Brassica rapa*. Clean reads Q30 (%) were larger than 89%, and clean reads ratio (%) was around 95% (Supplementary Table 2), indicating that the accuracy and quality of the sequencing data were sufficient for further analysis.

**FIGURE 8:**
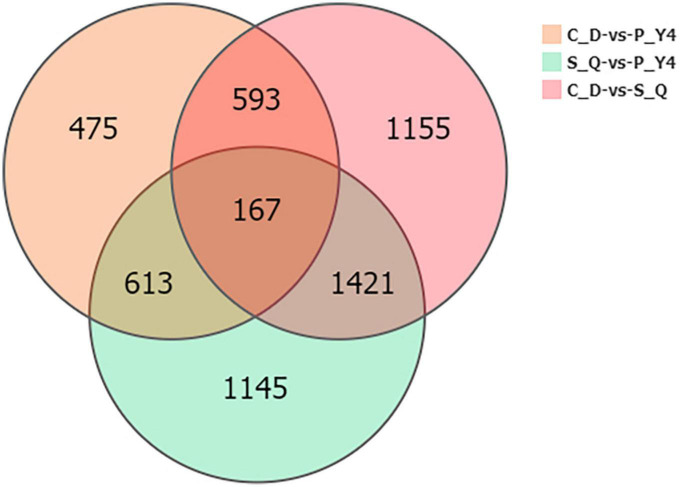
The Venn diagram of differentially expressed genes (DEGs) by comparing P-Y4 vs. C-D, S-Q vs. C-D, and P-Y4 vs. S-Q.

Next, the Venn diagram of differentially expressed genes (DEGs) was constructed by comparing P-Y4 vs. C-D, S-Q vs. C-D, and P-Y4 vs. S-Q (Figure 8). Among the genes with significant differences, the numbers of special DEGs were 1,145 in sample P-Y4 vs. S-Q, 475 in sample P-Y4 vs. C-D, and 1,155 in sample S-Q vs. C-D, which were involved in a Kyoto encyclopedia of genes and genomes (KEGG) pathway enrichment analysis. The result indicated that the differentially expressed genes related to the infection responses of primary zoospores and secondary zoospores were significantly enriched (Supplementary Table 3). The top one KEGG enrichment pathway was the plant-pathogen interaction (ko04626) by comparing P-Y4 with S-Q, phenylpropanoid biosynthesis (ko00940) by comparing P-Y4 with C-D, and plant hormone signal transduction (ko04075) by comparing S-Q with C-D, respectively.

**FIGURE 9:**
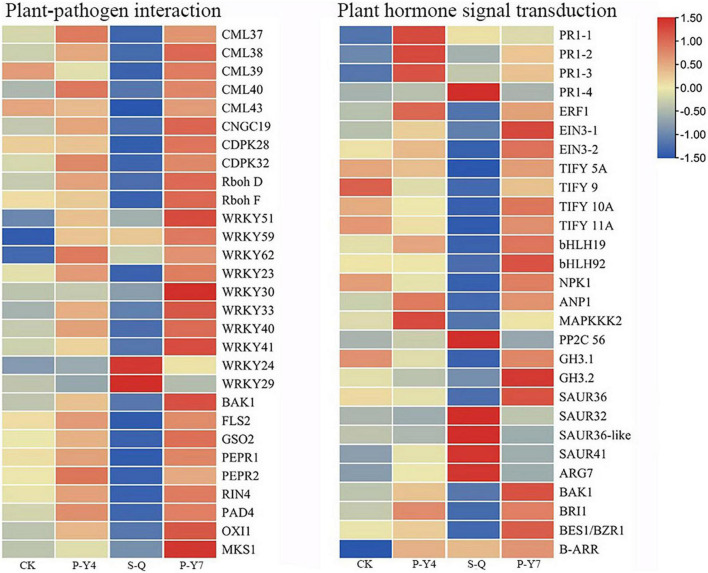
Heatmaps of differentially expressed genes in cabbage after primary zoospore and secondary zoospore inoculation. CK represents the control, P-Y4 represents gene expressions in cabbage at 4 days after resting spore inoculation, S-Q represents gene expressions in cabbage at 4 days after secondary zoospore inoculation, and P-Y7 represents gene expressions in cabbage at 7 days after resting spore inoculation.

In the plant-pathogen interaction pathway, the transcription levels of the pathogenesis-related proteins, *PR1* and *WRKY* (e.g., *WRKY51*, *54*, *59*, and *62*), were upregulated in *B. rapa* seedlings inoculated with both primary and secondary zoospores. The leucine-rich repeat receptor-like protein kinase *BAK1/BKK1*, *FLS2*, *GSO2*, and *PEPR 1/2*; calcium-dependent protein kinase *CDPK 32*; calmodulin-like protein *CaM/CML* (e.g., *12*, *37*, *38*, *38-like*, *39*, *39-like*, *40*, and *43*); cyclic nucleotide-gated ion channel *CNGC19*; *RPM1* (*At5g66900*); *RPM1* (disease resistance *RPP8-like*); disease resistance protein *RPS5* and RPM1-interacting protein *RIN4*; *Pti5* (ethylene-responsive transcription factor *ERF098*); *EFR* (*At2g24130*); *WRKY* (e.g., *23*, *30*, *33*, *40*, and *41*); and respiratory burst oxidase homology protein *Rboh* (e.g., *F*, *D*, *D-like*, and *G*) were not changed or upregulated in samples associated with resting spore inoculation but were downregulated in samples obtained from secondary zoospore inoculation. The expressions of *WRKY* (e.g., *20*, *24*, *28*, *29*, *38*, *59*, *70*, and *71*), *CML41*, and *EDS1B-lik*e were upregulated after inoculation with secondary zoospores (Figure 9). The results showed that most genes in the plant-pathogen interaction pathway were induced by inoculation with primary zoospores and suppressed by inoculation with secondary zoospores.

In the plant hormone signal transduction pathway, salicylic acid (SA), jasmonic acid (JA) and ethylene (ET) are important pathogen-response plant hormones. The expression of the SA marker gene, *PR1*, was upregulated, which indicated that the SA pathway was activated by inoculation with both primary zoospores and secondary zoospores. *EIN3* and *ERF1/2* in the ET pathway and *JAZ* and *MYC2* in the JA pathway were slightly upregulated or unchanged at 4 and 7 days after inoculation by primary zoospores but were suppressed at 4 days after inoculation by secondary zoospores.

In the cytokinin pathway, *B-ARR* (*PHL5-like*) was upregulated after inoculation by both primary zoospores and secondary zoospores. In the auxin pathway, the genes were not changed after inoculation with primary zoospores; however, 2 *GH3* and 2 *SAUR genes* were suppressed, and 9 *SAUR*, gibberellin pathway GID1 and TF, and ABA pathway PP2C genes were upregulated after inoculation with secondary zoospores alone (Figure 9; Supplementary Table 1). The data showed that the plant growth hormone pathway was mainly induced by secondary zoospore inoculation.

**FIGURE 10:**
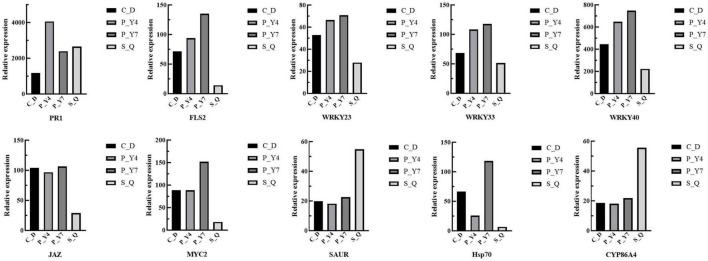
The gene expression was evaluated by qRT-PCR analysis to validate the RNA-seq results.

The expressions of 10 target genes were evaluated by qRT-PCR analysis to validate the RNA-seq results. The qRT-PCR results indicated high correlations among the selected genes (Figure 10).

## Discussion

The life cycle of *P. brassicae* is rather complicated and is generally divided into the primary infection phase in root hairs and the secondary infection phase in cortex tissue. McDonald et al. (2014) compared the roles of primary and secondary infections in both compatible and incompatible reactions. Here, we specified the roles in the life cycles of hosts and non-hosts inoculated with *P. brassicae* primary and secondary zoospores and focused especially on the infection differences.

### Primary zoospore infection was beneficial for establishing a compatible interaction between the host and *Plasmodiophora brassicae*

Obvious developmental asynchrony of *P. brassicae* occurred at 12 days in *B. rapa* inoculated with resting spores. A previous report indicated that cytoplasmic cleavages yielded massive amounts of uninucleate resting spores in cortical cells at more than 20 dpi (Liu et al., 2020a). Here, we first found that resting spores can be produced at 12 dpi. Meanwhile, limited numbers of zoosporangia were detected in root hairs and root epidermal cells after inoculation with secondary zoospores. The results demonstrate that primary infection influences the subsequent development of *P. brassicae*. A limited developmental process may be the reason that inoculation with secondary zoospores alone resulted in lower disease severity than inoculation with resting spores alone in canola (*Brassica napus*) (McDonald et al., 2014). The infectious difference between primary and secondary zoospores provides more evidence that the interaction of *P. brassicae* and the host mainly occurs during primary infection.

### Inoculation with secondary zoospores leads to more limited infection in the root hairs and epidermal cells of non-hosts than inoculation with primary zoospores

In spinach inoculated with resting zoospores alone, some zoosporangia groups formed, but when inoculated with secondary zoospores alone, we did not detect any zoosporangial clusters within the epidermal cells and root hairs. The infection frequencies of root hairs and epidermal cells with primary zoospore inoculation were higher than those with secondary zoospore inoculation. However, a previous report proved that in resistant hosts, inoculation with secondary zoospores resulted in much higher levels of cortical infection than inoculation with resting spores (McDonald et al., 2014), indicating that the response associated with non-host resistance was different from that of host resistance. The secondary zoospores from infected non-host spinach successfully infected cabbage and spinach, which indicated that *P. brassicae* was able to complete the primary infection in the non-host. This result was different from the previous observation that *P. brassicae* could initiate but not complete the primary infection in non-hosts (Liu et al., 2020b). Although root hair infection with *P. brassicae* appears to be not host-specific, repeating the zoosporangial stage was more obvious in cabbage seedlings with primary zoospore inoculation than those with secondary zoospore inoculation, which indicated that primary zoospores undertook an important interaction with plants. The results also indicated that intercropping *Brassica* crops with non-host crops was not suitable for managing *P. brassicae*.

### Most effectors were induced after inoculation with primary zoospores

To establish a successful infection, *P. brassicae* needs to either suppress the host resistance or fail to trigger host resistance. In either case, the response is likely triggered by effectors. Because the early infection stage is a key phase for the recognition and interaction between the host and *P. brassicae* (Zhao et al., 2017), we detected the gene expressions of *P. brassicae* after inoculations with primary and secondary zoospores. Although *P. brassicae* effectors were presented (Chen et al., 2019 and Pérez-López et al., 2020), the differential expressions and specific effectors released by primary zoospores and secondary zoospores were not compared. Here, among the 18 effectors identified, it was interesting to note that 14 effectors were induced after inoculation with primary zoospores. The initial recognition of *P. brassicae* may occur during the primary infection phase.

Only a few predicted effectors had conserved domains. The effectors Pb13 and Pb17 (chitin recognition protein), Pb18 (cystatin domain), Pb5 (thioredoxin), and Pb12 (ankyrin repeats) were induced by inoculation with primary zoospores. Considering the widespread nature of chitin perception in plants, pathogens could evolve strategies to overcome detection (Sánchez-Vallet et al., 2015), and the secretion of effectors Pb13 and Pb17 might provide cell wall protection or target host immune responses. Pb18 was identified as a cysteine protease inhibitor. Many previously identified plant pathogen effectors, namely, EPIC1 and EPIC2B, from the oomycete *Phytophthora infestans* (Song et al., 2009) and Pit2 from *Ustilago maydis* (Mueller et al., 2013), inhibit plant apoplastic cysteine proteases, an interaction that appears as a key for establishing compatibility (Lo Presti et al., 2015). *P. brassicae* might avoid host recognition or combat host defenses by secreting these effectors.

The expressions of effectors *Pb1* (alginate lyase) and *Pb4* (thaumatin family protein) were induced after inoculation with primary and secondary zoospores. Alginate lyase is effective in disrupting recalcitrant cell walls in *Laminaria digitata* (Costa et al., 2021), and plant cell wall-degrading enzymes secreted by biotrophic plant-pathogenic fungi facilitate their penetration of host plant cells (Gibson et al., 2011). When host cell walls are broken down, the effector may have a crucial role in zoospore infection of root hairs and epidermal cells.

The *P. brassicae* secondary infection effectors identified by Perez-Lopez did not include the chitin recognition protein, alginate lyase, thioredoxin, or thaumatin family protein. Our results indicated that primary zoospore infection in root hairs or epidermal cells acted as a major “battleground” with the host. Cysteine protease inhibitors and ANK-containing effectors were also identified during the late infection period (Pérez-López et al., 2020). This result indicated that these effectors played an important role in *P. brassicae* pathogenicity during the whole infection stage.

### Most genes in the plant-pathogen interaction pathway were induced after inoculation with primary zoospores but were suppressed by secondary zoospores

How does the host deal with a large number of effectors released by *P. brassicae* during the primary infection stage? Although the transcription data indicated the differentially expressed genes during the early or late stage of *P. brassicae* infection (Siemens et al., 2006; Päsold et al., 2010), no study has compared the host gene expression differences between primary and secondary zoospore infections. In the plant-pathogen interaction pathway, most genes were induced at 4 days after inoculation with resting spores (which corresponds to primary infection). Pathogen perception, the key PRR genes triggered by PAMPs, such as brassinosteroid insensitive 1-associated kinase 1 (*BAK1*), flagellin sensing 2 (*FLS2*), and EFR, which transduce signals that trigger PTI, showed slightly upregulated expressions after inoculation by primary zoospores but downregulated expressions after inoculation by secondary zoospores. Because inoculation with primary zoospores does not affect further development, PTI does not play a role in the interaction between *B. rapa* and *P. brassicae*, as previous reports have shown (Chen et al., 2016). After inoculation with primary zoospores, the CDPK signaling pathway and *Rboh* were also induced. Some identified effectors, such as Pb5, a thioredoxin protein that mediates redox signaling in plant immunity (Mata-Pérez and Spoel, 2019), may influence the host oxidation-reduction reaction.

Most of the defense pathway induction by primary zoospores was associated with a hypersensitive response (HR) or programmed cell death. Previous reports discovered that the damage was characterized by the degradation of cell walls in susceptible hosts but not in resistant hosts (Donald et al., 2008). The HR activated by primary zoospores might lead to cell wall breaks, which *favor P. brassicae* penetration and movement.

Otherwise, *WRKY* exhibited different expression patterns. Previous reports pointed out *WRKY 29*, *38*, *59*, and *62* upregulation and WRKY *23*, *33*, and *40* downregulation in resistant lines (Chen et al., 2016). Therefore, we suggest that WRKY 29, 38, 59, and 62 acted negatively and WRKY 33 acted positively in response to the presence of *P. brassicae*. The effectors released by *P. brassicae* primary zoospores might tend to avoid host defenses and establish compatible interactions.

### Most genes of the plant hormone signal transduction pathway were suppressed after inoculation with secondary zoospores

The expressions of the SA signal marker, PR1, showed significant increases in three samples, which was consistent with a previous study showing that *PR1* was upregulated in a susceptible line (Wei et al., 2021). The SA accumulations in the susceptible line were higher than those in the resistant line (Wei et al., 2021), which indicated that SA did not play a key role against *P. brassicae*. Inoculation with secondary zoospores inhibited *EIN3*, *JAZ*, and *MYC2* expressions in the JA signaling pathway. JA is believed to promote *P. brassicae* development and lead to host susceptibility (Xu et al., 2016; Li et al., 2018).

The expression levels of several genes in plant growth hormone pathways, such as BR pathway *BRI1* and *BES1/BZR1*, *cytokinins B-ARR* (*PHL5-like*), gibberellin pathway *GID1* and *TF*, ABA pathway *PP2C*, were changed, which indicates that these plant hormone pathways were regulated at an early stage of infection. Most IAA pathway-related genes were upregulated after inoculation with secondary zoospores (e.g., *auxin-induced protein 15A*, *SAUR22*, *32*, *36*, and *41*). By considering that *SAUR32* expressions were upregulated in a resistant line (Wei et al., 2021), we suggested that SAUR32 and several other SAURs might negatively regulate IAA levels. Previous studies have shown that auxin conjugate synthetases (*GH3*) are upregulated when auxin levels are higher (Jahn et al., 2013). Here, the downregulation of *GH3.1* and *GH3.2* might be related to low auxin levels after inoculation with secondary zoospores alone. These genes suppressed by inoculation with secondary zoospores might contribute to a susceptible reaction in the host at the subsequent infection stage. This might be one of the reasons why inoculation with secondary zoospores resulted in lower disease severity than inoculation with resting spores.

## Conclusion

**FIGURE 11:**
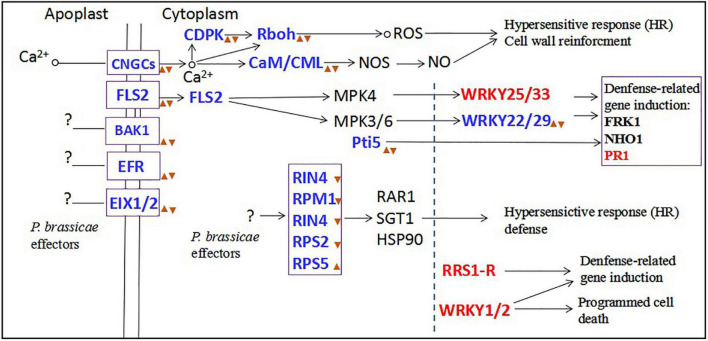
The plant-pathogen interaction pathway after primary zoospores or secondary zoospores infection. Red font represents the genes of upregulation expression by both primary and secondary zoospore infection compared with control. Blue fonts represent the genes of upregulation expression by primary zoospores infection compared with secondary zoospores. 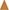 represent the genes of upregulation expression by primary zoospores infection compared with control. 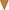 represent the genes of downregulation expression by secondary zoospores infection compared with control.

In our study, the results showed that inoculation with primary zoospores was more beneficial to the proliferation and development of *P. brassicae* and the formation of symptoms in plants than inoculation with secondary zoospores. To explore the reasons, we conducted transcriptome sequencing. Primary zoospores released many effectors, and their expression levels were significantly induced. Accordingly, in the hosts, we found that the calcium-mediated signaling pathway and PTI pathway were slightly induced by primary zoospores; however, obvious inhibition was observed when secondary zoospores were inoculated alone (Figure 11). The host defense induced after inoculation with resting spores fails against the invaded *P. brassicae.* These results indicated that the early interaction between the host and *P. brassicae* may be required to allow a fully compatible interaction and contributes to the susceptible reaction in the host at the subsequent infection stage. Secondary zoospores tend to better suppress the plant immune system. Our study at the molecular level further shows the roles of primary and secondary zoospores in pathogenesis. It is of great interest to understand how *P. brassicae* avoids or suppresses plant defense systems and facilitates its infection in hosts.

## Data availability statement

The datasets presented in this study can be found in online repositories. The names of the repository/repositories and accession number(s) can be found below: NCBI, accession numbers: PRJNA868504 and SRP392270.

## Author contributions

HY and WW conceived and designed the experiments. HY, YiZ, and YC performed the experiments of *P. brassicae* infection. HY and QS performed the experiments of RNA-Sequencing. QS and YaZ performed the experiments of identification of secreted proteins. YuZ and JD analyzed the data. YaZ and XW prepared the figures. SY critically revised the manuscript. All authors contributed to the article and approved the submitted version.

## Footnotes

1
http://brassicadb.org/brad/
